# The Effect of Simulated Patient Death on Learner’s Stress and Knowledge Retention: A Systematic Review and Meta-Analysis of Randomized Controlled Trials

**DOI:** 10.7759/cureus.43278

**Published:** 2023-08-10

**Authors:** Gunaseelan Rajendran, Ezhilkugan G, Aswin K, Sasikumar Mahalingam, Vimal Krishnan

**Affiliations:** 1 Emergency Medicine, Aarupadai Veedu Medical College and Hospital, Vinayaka Mission's Research Foundation, Puducherry, IND; 2 Emergency Medicine, Jawaharlal Institute of Postgraduate Medical Education and Research (JIPMER), Puducherry, IND; 3 Emergency Medicine, Indira Gandhi Medical College and Research Institute, Puducherry, IND; 4 Emergency Medicine, Kasturba Medical College, Manipal, IND

**Keywords:** systematic review and meta-analysis, simulated mortality, simulation design, knowledge retention, learner's stress, simulated patient death

## Abstract

Making the simulated patient die is one of the controversial decisions in healthcare simulation. Some experts believe that we should never make the manikin die as they believe the facilitator is deceiving the learners, whereas other groups of experts believe that there are advantages in making the simulated patient die as it provides a valuable learning experience to the learners, and it is as close to reality as possible. Hence, we undertook this review to know whether simulated patient mortality benefits the learners. A systematic literature search was performed in Embase, Scopus, PubMed Central, CENTRAL, MEDLINE, and Google Scholar. Randomized controlled trials assessing the learner’s stress and knowledge retention when the simulated patient dies were eligible for inclusion. Comparative intervention effect estimates obtained from meta-analyses were represented as pooled standardized mean difference (SMD) with a 95% CI. Six studies with 384 participants (learners) were eligible for the analysis. All the studies had some concerns when the risk of bias was assessed. In the simulated patient mortality group, the learners experienced higher stress as assessed compared to the group where the simulated patient survives. The two groups' pooled mean difference for anxiety and stress levels was 0.63 (0.17-1.09). Three out of five studies showed improved knowledge retention in the simulated mortality group, one showed no difference, and one showed decreased knowledge retention in the simulated mortality group. The stress response of learners when exposed to simulated mortality during a simulation session is higher than the simulated survival group. However, this increased stress response is processed by the students differently. Some students will thrive when increased stress is presented to them, while some students perceive it negatively. Thus, this increased stress response can lead to knowledge retention if the timing of the stress response happens mainly during debriefing for select students. The role of the facilitator is also important as skilled debriefers will be able to use this increased stress to their advantage to increase knowledge retention. Thus, simulated mortality can be used as an effective stressor for increasing knowledge retention during the debriefing phase for select students by a skilled debriefer. This study would aid the simulation policymakers, simulation faculties, and simulation researchers in the impact of simulated patient death and learners’ stress response. If the simulation scenario is designed well with robust pre-briefing, this increased stress response can enhance learning and knowledge retention during debriefing.

## Introduction and background

Simulation is one of the most powerful instructional methods used in medical education to teach students in the healthcare domain. David Gaba et al. defined simulation as a contextual event in space and time conducted for one or more purposes in which people interact in a goal-oriented fashion with each other, with technical artifacts (the simulator) and the environment (including relevant devices) [1]. Simulation provides the learners with a safe but close to the real learning environment, thus translating directly to patient safety. Simulation is an instructional method where learning happens through experiential learning and reflection. The technique of simulation includes pre-brief, the actual scenario, and debriefing. In the pre-brief, the facilitator establishes a psychologically safe environment, suspends the disbelief, and gets a fiction contract in place. Then the actual scenario happens where the facilitator takes the patient through a real-life or fictional scenario, and the learners have to manage the simulated patient, either a manikin or a standardized patient. This will be followed by debriefing. This phase of simulation is where maximum learning happens. Although healthcare simulation is well-published, the exponential technical growth has exposed newer challenging and controversial areas. One such controversial area is the death of the simulated patient [2-7]. Leighton classified simulated patient death into three types: expected death, unexpected death, and death due to actions or inactions [8]. An expected death is when the learners receive the information before the scenario begins and are prepared for it. An unexpected death is when the learners are unaware of the case scenario but is intentionally introduced by the facilitator during the simulation. Death due to action or inaction is when the simulated patient dies because of some act done by the learners, and both the learners and the facilitators are surprised by it [8]. An example would be when the learners administer a medication's wrong dose (potentially lethal dose).

Evidence on the educational benefit for the learners, when the simulated patient dies, is controversial. Some authors believe that the simulator should never be made to die, while some groups of authors allow the practice. The pros of making the simulator die are (1) providing valuable learning experiences that would not be available in routine clinical environments, (2) the ability to learn in a safe environment, (3) the ability to reflect on personal feelings about death, and (4) decreases fear, anxiety, and feeling of inadequacy for the learners [5]. However, the simulated patient death can distract the learners from the main objectives of the simulation session and may psychologically demotivate them from participating in future simulation activities [5]. The main concern regarding simulated patient death is the psychological safety of the learners. Simulated patient death is believed to create stress which affects learning and memory. The unexpected death of the manikin (patient) during the simulation may also cause negative feelings toward the simulation. Another concern about simulation sessions involving the death of a simulator is debriefing. Debriefing is the essence of simulation and experiential learning. There are many models of debriefing. One commonly used model is the general adaptation syndrome (GAS) model and the ABCDE (avoid shaming/personal opinions, build a rapport, choose a communication approach, develop a debriefing content, ensure the ergonomics of debriefing) model [9]. In the GAS model, we gather information on how the participants performed during the simulation. The HOW and WHAT questions can achieve this [10]. We then analyze the information for what all went well and what things could have been improved. A summary phase will follow this. Many other models of debriefing also exist [11]. Only a skilled facilitator would be able to facilitate the debriefing session in the right direction so that the learning objectives are explored and discussed [5]. Ensuring the psychological safety of the learner is another challenge for the facilitator.

To understand better the impact of simulated patient death on the learner’s stress and knowledge retention, we conducted this systematic review of randomized controlled trials.

## Review

Design

We conducted a systematic review of randomized controlled trials. We followed the Preferred Reporting Items for Systematic Reviews and Meta-Analyses (PRISMA) checklist for reporting systematic reviews incorporating meta-analyses for reporting our review.

Research question

Does the simulated patient's death, compared to the simulated patient's survival, negatively affect the learner’s emotions and knowledge retention during a team simulation activity?

Inclusion criteria for the studies

We included studies conducted among adults above 18 years of age who are students, interns, or clerks in the healthcare profession and healthcare workers who participated in team simulation activities. We included the participants irrespective of their seniority level or prior experience in simulation. We included studies that assessed learners' knowledge retention, emotional response, stress, or anxiety level when exposed to simulated mortality. We included only randomized controlled trials. Studies reported as full text were included, while studies published with only abstract or unpublished data were excluded.

Search strategies

We extensively searched MEDLINE, Embase, Scopus, and Google Scholar databases. The following MeSH terms and free text terms were used in the PubMed Central search engine by two independent investigators (GR, EG) in various combinations with the randomized controlled trial filter: “Patients,” “Death,” “Simulation,” “Simulated Patient Death,” “Simulated Mortality,” and “Patient Simulation.” Similar terms were used in Embase, Scopus, and Google Scholar for literature searches of published studies. There were no restrictions on the date of publication, country, and language. We also searched the primary selected articles' references for additional relevant articles to be included in the review.

Selection of studies

Two independent investigators (GR and EG) performed the literature search and screened the title, abstract, and keywords. The full-text article was obtained for the relevant studies, and conference abstract articles were excluded. Further screening of the full text of the retrieved articles was done independently by two investigators (GR and EG) to select the studies satisfying the inclusion criteria. Any disagreements during this selection process were resolved through consensus or consultation with a third investigator (AK). The quality of the selection process was also monitored by the third investigator (AK).

Data extraction

The primary author (GR) extracted data from the selected studies for the authors' names, year of publication, type of study, number of randomized groups, and sample size in each group. Further, the learners' experience level and their experience in the simulation were also extracted. The outcome of each activity was also extracted. The extracted outcome included the state-trait anxiety inventory (STAI) score, time to request diagnostic information, time to treatment, anesthetist's non-technical skills scale, an eight-item scale containing bipolar-opposite descriptors of emotion based on the semantic structure of emotion proposed by Feldman Barrett and Russell, heart rate response, salivary cortisol level, and salivary dehydroepiandrosterone levels.

Risk of bias assessment of the included studies

The risk of bias assessment was carried out using the RoB2 tool published by Cochrane Collaboration [12]. Two independent investigators (GR and EG) analyzed the five domains in the RoB2 tool, namely bias arising from the randomization process, bias due to deviations from intended interventions, bias due to missing outcome data, bias in the measurement of outcome, and bias in the selection of reported results and overall risk of bias.

Results

**Figure 1:**
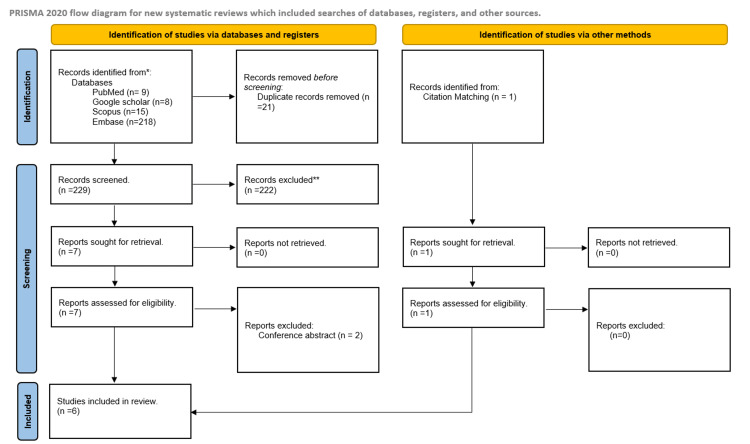
PRISMA flow diagram of the selection of studies PRISMA: Preferred Reporting Items for Systematic Reviews and Meta-Analyses

In total, 250 studies were identified using the above search strategy. Twenty-one duplicate studies were removed. Two hundred twenty-nine studies were screened based on the title, abstract, and keywords. Seven studies were included to retrieve full texts; two were found to be conference abstracts excluded. We reviewed the references and citations of these studies and included one more relevant study. Finally, we analyzed data from six studies with 384 participants [13-18]. Figure 1 shows the PRISMA flow diagram.

Table 1 lists the characteristics of the study that were included in the review. Four out of the six studies were carried out in the USA. Five of the six studies were uniprofessional, and one study was interprofessional. In three of the studies, the participants were anesthesiology residents, and in two of the studies, the participants were medical students. The studies were conducted between 2014 and 2019. The sample size ranged from 12 to 61 participants. The mean age group of the participants in the study was 25 years to 30 years. The stress response was measured using the mean STAI score in three studies. One study measured the stress response using heart rate response and salivary cortisol level. In another study, the emotional response was measured using an eight-item scale containing bipolar opposite descriptors of emotion based on the semantic structure of emotion proposed by Feldman Barrett and Russell.

**Table 1:** Characteristics of the included studies * Independent mortality indicates the simulated patient will die and the participant will not have consultant support. Supported mortality indicates the simulated patient will die, and the participant will have consultant support. RCT: randomized controlled trial, DHEA: dehydroepiandrosterone, ANTS: anesthesia non-technical skills, STAI: state-trait anxiety inventory

Sl No	First Author and Year	Country	Study Design	Study Participants	Outcomes Reported	Age Group (Years)	Number of Randomized Groups	Sample Size in Simulated Patient Survival	Tools Used
1	Burnett et al., 2019 [13]	USA	RCT, observer-blinded	First-year residents in anesthesiology	Time to request diagnostic information. Time to treatment	27 (independent mortality not possible), 27 (independent mortality possible), 27 (supported mortality not possible), 27 (supported mortality possible)*	4	12 (independent mortality not possible), 12 (independent mortality possible), 12 (supported mortality not possible), 12 (supported mortality possible)	Nil
2	DeMaria et al., 2016 [14]	USA	RCT	Medical students in years 2-4	1. Heart rate response, 2. salivary cortisol, 3. salivary DHEA	25.1 (survival group), 26.5 (death group)	2	13 (survival group), 13 (death group)	ACLS checklist
3	Fraser et al., 2014 [15]	Canada	RCT	Final-year medical students	Emotional assessment and cognitive load		2	55 (unexpected death), 61 (patient survival)	1. Nedelsky method, 2. emotional assessment by Feldman Barrett and Russell tool, 3. cognitive load assessment bypass and Van Merrienboer tool
4	Goldberg et al., 2015 [16]	USA	RCT	First-year residents in anesthesiology	Mean ANTS score and mean STAI score	29 (independent group), 28.5 (supervised group)	2	12 (independent group), 12 (supervised group)	1. ANTS
5	Goldberg et al., 2017 [17]	USA	RCT	PGY-2 residents in anesthesiology	Mean ANTS score and mean STAI score	29 (never death), 30 (always death), 29 (variable death)	3	17 (never death), 18 (always death), 15 (variable death)	1. ANTS, 2. STAI (state portion), 3. trauma rating, 4. helpfulness rating, 5. engagement rating
6	Phillipon et al., 2016 [18]	France	A cluster-randomized trial	Healthcare assistants, nurses, medical students, and residents	STAI - state anxiety score	Mean - 30 in the mortality group and 29 in the survival group	2	63 (death group), 57 (life group)	1. STAI - state-trait anxiety score, 2. Likert scale

The risk of bias assessment was conducted using the RoB2 tool and reported in Table 2. The overall risk of all six studies was "some concerns." All six studies had low risk in the randomization process (Domain 1) and deviation from the intended intervention (Domain 2). Three of six studies had some risk in the missing outcome data domain (Domain 3). Two out of six studies had some risk in the measurement of the outcome domain (Domain 4). All six studies had some risk in selection in the reported result (Domain 5).

**Table 2:** Risk of bias for the included studies * Some concerns mean that the study is judged to raise "some" concerns in at least one domain for this result but not at high risk of bias for any domain.

Article and year	Domain 1 (risk of bias arising from the randomization process)	Domain 2 (risk of bias due to deviations from the intended interventions)	Domain 3 (risk of bias due to deviations from the intended interventions)	Domain 4 (risk of bias due to missing outcome data)	Domain 5 (risk of bias in the measurement of the outcome)	Overall
Burnett et al., 2019 [13]	Low	Low	Low	Low	Some	Some concerns*
DeMaria et al., 2016 [14]	Low	Low	Some	Low	Some	Some concerns
Fraser et al., 2014 [15]	Low	Low	Low	Some	Some	Some concerns
Goldberg et al., 2015 [16]	Low	Low	Low	Low	Some	Some concerns
Goldberg et al., 2017 [17]	Low	Low	Some	Some	Some	Some concerns
Philippon et al., 2016 [18]	Low	Low	Some	Low	Some	Some concerns

A narrative synthesis of the results

We analyzed six studies with 384 participants in the study. Philippon et al. reported a reduction in mean STAI score and anxiety in both the simulated mortality and survival group after one month of simulation with a life-threatening scenario. Burnett et al. reported that time to treatment was less in the group where death was possible, and the total treatment score was also better in the group where death was possible [13]. DeMaria et al. also reported no readily detectable difference in stress response between simulated mortality and survival groups [14]. However, Goldberg et al. (2015) [16] & Goldberg et al. (2017) [17] reported an increase in the stress and anxiety levels measured by STAI score, which was statistically significant. A similar observation was made by Fraser et al., who measured the stress response using an eight-item scale [15]. Three studies reported no difference in stress levels between the two groups, and three reported significant differences.

**Figure 2:**
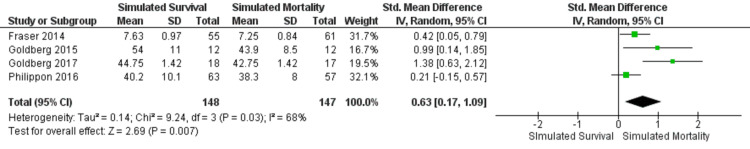
Forest plot of various studies [15-18]

A meta-analysis was performed with four studies that reported the mean using STAI scores and an eight-item scale. We used the RevMan 5 software (Cochrane, London, England) to create the forest plot. We also used the random effects model and SMD as the outcome variables were continuous. The two groups' pooled mean difference for anxiety and stress levels was 0.63 [0.17-1.09] (Figure 2). This showed increased stress levels when the participants experienced simulated mortality compared to simulated survival. However, the studies had substantial heterogeneity as the I2 value was 68% with a p-value of 0.03. We also performed a sensitivity analysis which is shown in Table 3. The sensitivity analysis doesn't affect the overall pooled estimate suggesting that the final estimate is robust for any changes. We also performed a GRADE analysis for this outcome. We double-downgraded the randomized controlled trials as there were some concerns about the risk of bias, and there is substantial heterogeneity. Hence, the final grading of the evidence stating that the stress levels increase in the simulated mortality group is of low evidence.

**Table 3:** Sensitivity analysis for the association between simulated mortality and learner’s stress SMD: standardized mean difference

Overall pooled SMD	0.63 [95% CI: 0.17-1.09]
Pooled SMD after removing Fraser et al., 2014 study [15]	0.80 [95% CI: 0.01-1.59]
Pooled SMD after removing Goldberg et al., 2015 study [16]	0.56 [95% CI: 0.05-1.08]
Pooled SMD after removing Goldberg et al., 2017 study [17]	0.39 [95% CI: 0.08-0.71]
Pooled SMD after removing Phillipon et al., 2016 study [18]	0.86 [95% CI: 0.23-1.49]

Fraser et al. reported reduced knowledge retention in the simulated mortality group. They were less competent in managing a patient than the simulated survival group [15]. DeMarai et al. reported no statistically significant difference in knowledge retention when analyzed after six months of simulation in both simulated mortality and survival group [14]. However, Burnett et al. observed reduced treatment time in the simulated mortality group compared to the simulated survival group [13]. On two occasions, Goldberg et al. observed that the learners' performance was better in the simulated mortality group when assessed six weeks and six months later, respectively, compared to the simulated survival group [16-17]. In conclusion, three out of five studies showed improved knowledge retention in the simulated mortality group, one showed no difference, and one showed decreased knowledge retention in the simulated mortality group.

Discussion

Our systematic review and meta-analysis showed that the stress response in the simulated mortality group is higher than in the simulated survival group. The increased stress response could be directly attributed to the simulated patient mortality, as three of the four studies we took for meta-analysis had the same scenario in both groups. Only one study had different scenarios for different teams of participants. The other confounders for the stress response in both groups were case fidelity and agency, as described by Gaba and Aaron [19]. In this context, the degree to which the instructor connects the students' actions to the clinical result shown by the mannikin is referred to as case fidelity. There was a substantial lack of case fidelity in both groups, as the learners' action was deliberately nullified. The concept of agency refers to the capacity of the learners to act in a particular way that results in specific results. For example, administering insulin and dextrose should reduce the serum potassium level, and the learner will administer insulin dextrose when faced with a simulated patient with hyperkalemia. However, in the simulated mortality group, this action would not have happened, which could also contribute to the increased stress of the learners. The learners would have known whether the manikin would live or die because of the combined effects of the case fidelity and agency. This might have eliminated the blinding process.

In our systematic review for knowledge retention, varied responses were noted. Three out of five studies showed improved knowledge retention in the simulated mortality group, one showed no difference, and one showed decreased knowledge retention in the simulated mortality group. This varied response could be due to the effect of stress on learning and memory. Yerkes and Dodson showed an inverted U-shaped relationship between stress and learning [20]. When there is no or minimal arousal (stress), there is no improvement in performance, and when there is very high arousal (stress) also, there is no improvement in performance. They showed that optimal arousal (stress) led to optimal performance. However, the term optimal stress will vary with individuals. The level of stress for one person may overwhelm another. Due to the vulnerability to stress, some people perform better under pressure than others, which could have been one reason for the varying results between these studies.

Vogel and Schwabe reported that stress around the time of learning could enhance memory formation [21]. Joëls et al. observed that stress would enhance learning and memory when the stress is experienced around when the information needs to be remembered [22]. Stress and learning have a time-bound relationship. This is mainly due to the release of corticosteroids which in the short term facilitates the strengthening of contacts involved in memory formation but, in the long term, initiates a gene-mediated signal that will suppress any information unrelated to the event reaching the same areas hours later. Simulation is an activity where the learning process mainly occurs during debriefing, which happens immediately after the simulation activity. Hence, stress during this phase might have potentiated the learning process in the three studies.

Further, the individual response to and handling stress may also vary depending on the individual's personality traits. Individuals with neuroticism personalities rely on disengagement as a means of coping mechanism, which results in disengagement during debriefing [23]. Thus, the actual effect of simulated mortality on knowledge retention cannot be truly studied as it is affected by the timing of stress and the individuals' response to it.

Limitations

All the studies were done in the Western setting, which was a significant limitation of our review. Furthermore, we analyzed the stress response of the individuals when exposed to a simulated patient death during a team simulation. However, the individuals' ability to cope with stress was not analyzed.

## Conclusions

The learners experienced slightly higher stress when the simulated patient died during a simulation session. However, this increased stress response is processed by the students differently. Some students will thrive when increased stress is presented to them, while some students perceive it negatively.

Thus, this increased stress response can lead to knowledge retention if the timing of the stress response happens mainly during debriefing for select students. The role of the facilitator is also important, as skilled debriefers will be able to use this increased stress to their advantage to increase knowledge retention. Thus, simulated mortality can be used as an effective stressor for increasing knowledge retention during the debriefing phase for select students by a skilled debriefer.
